# Incidental T1a Gallbladder Cancer with Signet Ring Cell Carcinoma Following Laparoscopic Cholecystectomy: A Case Report

**DOI:** 10.70352/scrj.cr.24-0078

**Published:** 2025-02-14

**Authors:** Yoshihito Kitamura, Masakazu Hashimoto, Ryo Nagao, Makoto Shinohara, Keigo Nakashima, Yui Hattori, Michinori Hamaoka, Masashi Miguchi, Toshihiro Misumi, Nobuaki Fujikuni, Satoshi Ikeda, Yasuhiro Matsugu, Takashi Nishisaka, Hideki Nakahara

**Affiliations:** 1 Department of Gastroenterological Surgery, Hiroshima Prefectural Hospital, Hiroshima, Hiroshima, Japan; 2 Department of Pathology and Laboratory Medicine, Hiroshima Prefectural Hospital, Hiroshima, Hiroshima, Japan

**Keywords:** signet ring cell carcinoma, T1a cancer, laparoscopic cholecystectomy, incidental gallbladder cancer, radical second resection

## Abstract

**INTRODUCTION:**

Signet ring cell carcinoma (SRC) of the gallbladder is a rare type of gallbladder cancer. We report a case of SRC of the gallbladder that was characterized by the diffuse presence of SRC on the gallbladder mucosa and diagnosed after cholecystectomy.

**CASE PRESENTATION:**

A 40-year-old man was referred to our department with upper abdominal pain and vomiting. Based on the findings of blood tests, computed tomography, and magnetic resonance imaging, acute cholecystitis was suspected, and emergency laparoscopic cholecystectomy was performed. Intraoperative findings showed mild inflammation. Although the tumor remained within the mucosa, tumor cell infiltration was suspected at the edge of cystic duct pathologically. Although additional endoscopic ultrasound and endoscopic retrograde cholangiography showed that horizontal extension into the residual cholecystic duct was suspected, there was no evidence of invasion into the common bile duct, lymph node metastasis, or distant metastasis. One and a half months after cholecystectomy, the patient underwent extrahepatic bile duct resection, lymph node dissection, and bile duct jejunal anastomosis. The postoperative course was uneventful, and the patient was discharged on the 10th postoperative day. Postoperative pathological analysis showed no obvious residual tumor tissue in the common bile duct or choledochal duct margins, and no metastasis in the submitted lymph nodes. Based on the above, a diagnosis of pT1aN0M0, pStage IA SRC was made. As no lymph node metastasis was observed, it was decided to follow up the patient without initiating postoperative chemotherapy, and the patient has been recurrence-free for 12 months after surgery.

**CONCLUSIONS:**

We describe an incidentally discovered case of intramucosal SRC diffusely spreading throughout the gallbladder after cholecystectomy for acute cholecystitis.

## Abbreviations

SRC
: signet ring cell carcinoma
PAS
: periodic acid-Schiff
CT
: computed tomography
MRCP
: magnetic resonance cholangiopancreatography

## INTRODUCTION

Gallbladder cancer is often diagnosed through pathological examination after cholecystectomy. The histological subtype of gallbladder cancer is mainly papillary adenocarcinoma or tubular adenocarcinoma, while signet ring cell carcinoma (SRC) is rare.

## CASE PRESENTATION

**Fig. 1:**
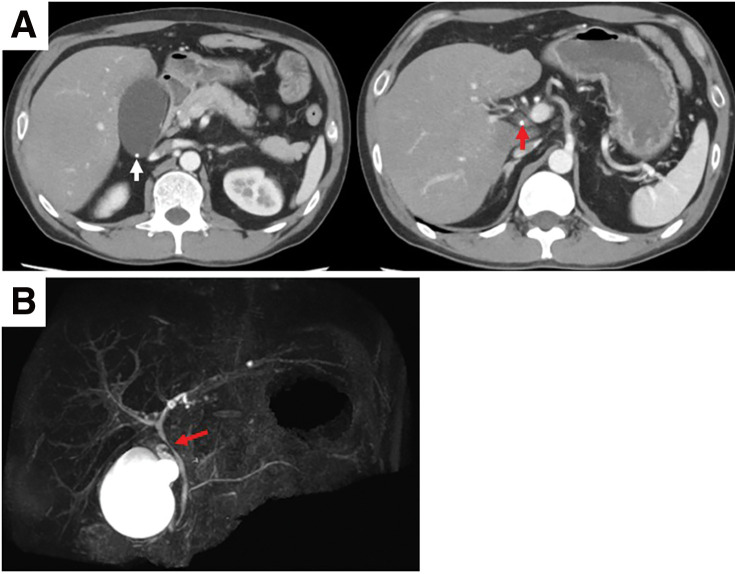
The image findings after the initial surgery. (**A**) Contrast-enhanced computed tomography showed an enlarged gallbladder with slightly enhancement of the gallbladder bed. The white arrow shows gallstones in the gallbladder. The red arrow shows gallstones incarceration in the cystic duct. (**B**) On magnetic resonance cholangiopancreatography, the biliary tract abnormality was not presented, and the common bile duct stones were not detected. The red arrow shows the gallstone pressing on the common bile duct.

A 40-year-old man presented to our hospital with upper abdominal pain and vomiting. Blood testing revealed a white blood cell count of 8900 × 10^9^ and a C-reactive protein level of 0.35 mg/dL. Additionally, the aspartate aminotransferase, alanine aminotransferase, and lactate dehydrogenase, and gamma-glutamyl transferase levels were 260, 150, 342, and 1309 U/L, respectively. Meanwhile, the total bilirubin level was not elevated. Contrast-enhanced computed tomography (CT) revealed that the gallbladder was enlarged, gallstones were incarcerated in the cystic duct, and the gallbladder bed was slightly enhanced. Severe inflammation around the gallbladder was not detected by CT (**Fig. 1A**). On magnetic resonance cholangiopancreatography (MRCP), there was no biliary tract abnormality, common bile duct stones were not detected, and the gallstones were pressing on the common bile duct (**Fig. 1B**). Laparoscopic cholecystectomy was then performed. Intraoperatively, the cystic duct and cholecystic artery were clipped, and the gallbladder was removed without damaging its wall. A drain tube was then placed on the gallbladder bed. Grossly, gallstones approximately 1 cm in diameter were found in the gallbladder. Postoperatively, the total bilirubin level increased to 4.3 mg/dL, but gallstones were not detected in the common bile duct on MRCP. Flopropione was administered, and the total bilirubin level gradually decreased. Five days after surgery, the patient was discharged.

**Fig. 2:**
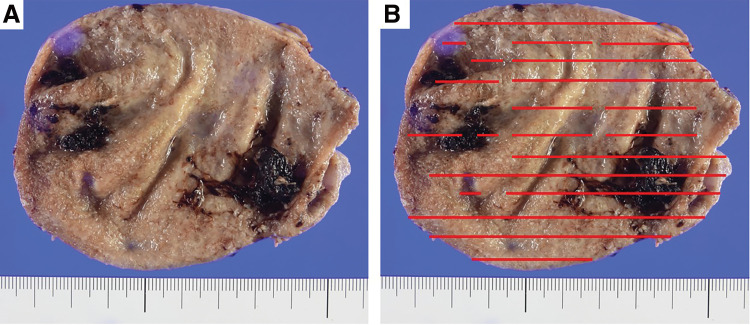
Gross pathological findings of the gallbladder. (**A**) The gallbladder mucosa appears glossy under the naked eye, but is irregularly granular, and dark red tissue that appear to be bleeding or degeneration are seen in a map-like pattern. (**B**) Red lines show sites of suspected tumor cell infiltration.

**Fig. 3:**
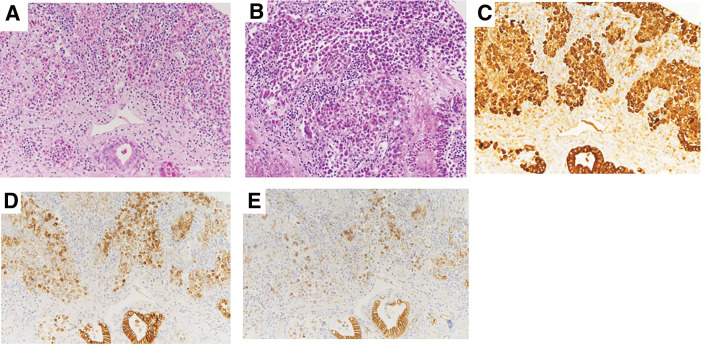
Special and immunological staining of the gallbladder. (**A**) Periodic acid-Schiff positive intracytoplasmic mucus, magnification ×200. (**B**) Periodic acid-Schiff-diastase positive intracytoplasmic mucus, magnification ×200. (**C**) Cells were positive for cytokeratins AE1; CK10, CK12, CK14, CK15, CK16, CK19/AE3; CK1, CK3, CK4, CK5, CK6, CK7, and CK8, magnification ×200. (**D**) Cells showed weaker expression for E-cadherin, magnification ×200. (**E**) Cells showed weaker expression for β-catenin, magnification ×200.

**Fig. 4:**
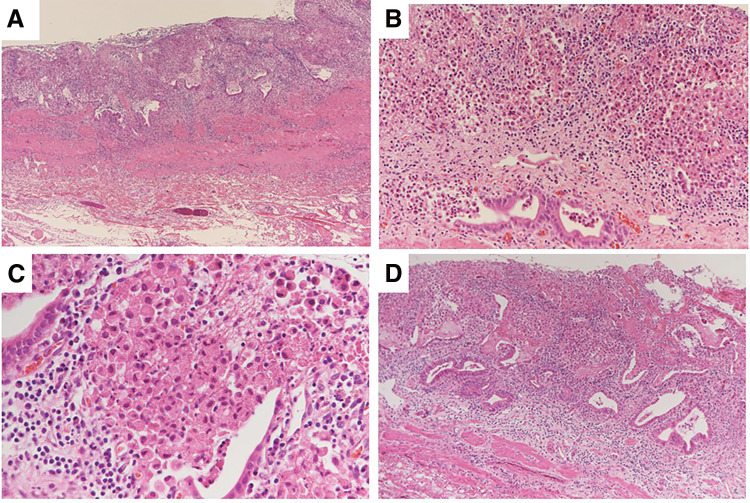
Pathological findings of the gallbladder upon hematoxylin and eosin staining. (**A**) Signet ring cell carcinoma (SRC) cells exist diffusely within the gallbladder mucosa, hematoxylin and eosin stain, magnification ×40. (**B**) SRC cells exist diffusely within the gallbladder mucosa, hematoxylin and eosin stain, magnification ×200. (**C**) SRC cells are oval-shaped, contain cytoplasmic mucus, and have an unevenly distributed nucleus, magnification ×400. (**D**) SRC cells at the margin of the cystic duct, magnification ×100.

Considering the pathological findings of the gallbladder, the mucosa appeared glossy under the naked eye; however, its structure was irregularly granular, and dark red tissues, indicative of bleeding or degeneration, were seen in a map-like pattern (**Fig. 2**). Furthermore, tumor cells were positive on periodic acid-Schiff (PAS) and PAS-diastase staining (**Figs. 3A** and **3B**). Immunological staining showed that these cells were also positive for cytokeratin (AE1/AE3), and weekly positive for E-cadherin and β-catenin (**Fig. 3**). Tumor cells were negative for synaptophysin, CD56, and chromogranin A. The pathology results after the initial surgery showed diffuse SRC within the gallbladder mucosa (**Fig. 4**).

**Fig. 5:**
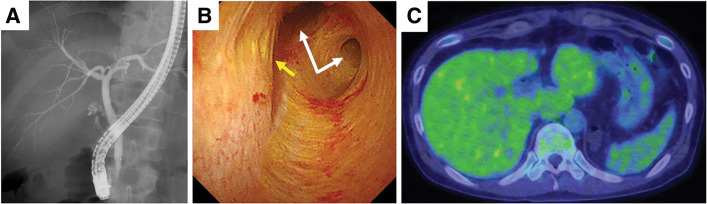
Additional examinations before the second surgery. (**A**) Endoscopic retrograde cholangiopancreatography showed neither stenosis of the common bile duct nor an anomalous pancreaticobiliary ductal junction. (**B**) Peroral cholangioscopy showed no evidence of invasion into the common bile duct. The white arrows show the hepatic bile duct. The yellow arrow shows the junction of the cystic duct. (**C**) Positron emission tomography showed no evidence of lymph node metastasis or distant metastasis.

Tumor cell infiltration was suspected at the margin of the cystic duct; therefore, we underwent additional resection. Although endoscopic ultrasound, endoscopic retrograde cholangiopancreatography, and positron emission tomography showed horizontal extension into the residual cholecystic duct was suspected, there was no evidence of invasion into the common bile duct, lymph node metastasis, or distant metastasis (**Fig. 5**). In addition, the CT scan did not reveal any obvious tumors in the digestive tract. Moreover, upper and lower endoscopic examinations were also performed; however, no tumorous lesions were found. Thus, the patient was diagnosed with primary gallbladder signet ring cell carcinoma. Approximately one and a half months after cholecystectomy, the patient underwent open gallbladder bed resection, extrahepatic bile duct resection, lymph node dissection, and bile duct jejunal anastomosis. The postoperative course was uneventful, and the patient was discharged on the 10th postoperative day. The pathology results after the second surgery showed no obvious residual tumor tissue in the common bile duct or choledochal duct margins and no metastasis in the submitted lymph nodes. We diagnosed SRC of the gallbladder as pT1aN0M0 pStage IA. As no lymph node metastasis was observed, the patient was followed up without administering postoperative chemotherapy, and the patient has been recurrence-free for 10 months after surgery.

## DISCUSSION

Our case is unique as the cancer was diagnosed SRC of the gallbladder at an early stage despite diffusely invading the mucosa. Gallbladder cancer is the fifth most common malignant neoplasm in the gastrointestinal tract and the most common malignancy in the biliary tract.^1)^ Approximately 1%–2% of patients undergoing surgery for cholelithiasis are diagnosed with cancer postoperatively.^2)^ Gallbladder cancer has a poor prognosis; in papers published before the 2000s, its 5-year survival rate was approximately 32% even if the disease remains within the mucosa, while the 1-year survival rate was 10% if it invades outside the mucosa.^3–5)^ However, the survival rate of gallbladder cancer has been improving. National Cancer Database of the American College of Surgeons report showed the following 5-year survival rates for patients with gallbladder cancer by stage: 80% for stage 0, 50% for stage I, 28% for stage II, 8% for stage IIA, 7% for stage IIB, 4% for stage IVA, and 2% for stage IVB.^6)^ In cases of T1a gallbladder cancer, the 5-year survival rate is 70%–100%.^7)^ Gallbladder cancer does not manifest with specific symptoms; thus, it is difficult to diagnose it in its early stages.

Risk factors for gallbladder cancer include gallstones, chronic cholecystitis, primary sclerosing cholangitis, gallbladder polyps, anomalous pancreaticobiliary ductal junction, obesity, metabolic syndrome, and diabetes mellitus.^2,6,8)^ Cholelithiasis is an important risk factor for gallbladder cancer, with 70%–90% of patients having a history of cholelithiasis.^6,9)^ Additionally, gallstones >3 cm increase the risk of gallbladder cancer by a factor of 9.2–10.1 compared to gallstones <1 cm.^6,10)^ Although the mechanism by which cholelithiasis can result in gallbladder cancer remains unclear, chronic epithelial irritation and mucosal damage may be involved. In our case, gallstones were present, but they were <3 cm. Anomalous pancreaticobiliary ductal junction is another important risk factor for gallbladder cancer. The junction of the pancreatic and bile ducts is not covered by the duodenal papillary sphincter muscle, causing backflow of pancreatic juice and bile. As the pressure in the pancreatic duct is usually higher than that in the bile duct, pancreatic juice backflows through the bile duct, inducing gallbladder mucosal damage and inflammation that lead to carcinogenesis.^11)^ Approximately 10% of patients with gallbladder cancer have this anatomic abnormality, which was not observed in our patient.

Adenocarcinoma is the most common histological type of gallbladder cancer (80%–95%), while SRC is rare.^2,12)^ Risk factors specific to SRC remain unclear, but patients with SRC are older, and most are women, when compared to patients with non-SRC cancer.^12)^ A recent report comparing the prognosis of SRC to that of non-SRC showed a 5-year survival rate of 7.2% for SRC and 13.2% for non-SRC, suggesting that the prognosis may be worse for patients with SRC.^12)^ This report also showed that SRC is associated with a lower differentiation, more advanced stage, lymph node metastasis, distant metastasis, and advanced anomalous pancreaticobiliary ductal junction compared to non-SRC cancer. In our case, there was diffuse mucosa-wide infiltration of indurated cells, which is a very unusual pathology.

SRC commonly occurs in gastric cancer. The prognosis for SRC of the stomach is better than that of gastric adenocarcinoma if it is stage Ia; however, if it is stage III, the prognosis is worse.^13)^ While SRC of other organs, such as the pancreas and colon, is more common in men, SRC of the gallbladder is more common in women, with a male: female ratio of 0.30.^12)^

Treatment of gallbladder cancer involves surgery. For T1a tumors, as the likelihood of residual disease or lymph node metastasis after cholecystectomy is extremely low (1.8%), and gallbladder bedside resection and lymph node dissection are unnecessary. Simple cholecystectomy is sufficient^.7)^ Meanwhile, for T1b tumors, there is an 11% risk of lymph node metastasis and a higher chance of postoperative lymph node recurrence compared with T1a cancer; therefore, regional lymph node dissection should be considered.^7)^ In our case, additional resection was performed because tumor cell infiltration was suspected at the margins of the cystic duct. If residual tumor is present, margin-free surgery should be performed. However, there is no consensus on whether lymph node dissection or gallbladder bed resection should be performed when the cystic duct margin is positive for SRC. In our case, the preoperative examinations did not show any obvious lymph node metastasis or liver infiltration. However, the cystic duct margin was positive, and considering the possibility of extension into the common bile duct, the patient’s age, and risk of recurrence as well as due to the poorer prognosis of SRC compared to adenocarcinoma, we performed gallbladder bed resection, extrahepatic bile duct resection, and lymph node dissection. Although this tumor was an early-stage cancer, initial surgery revealed a positive margin of the cystic duct, raising concerns about the potential risk of peritoneal dissemination. Adjuvant S-1 after an additional surgery might improve the prognosis.^14)^ Nevertheless, the recommended surgical procedure and adjuvant therapy for cases similar to ours remain unclear, and further cases are warranted to better clarify the appropriate treatment approach.

## CONCLUSIONS

We experienced a case of SRC of the gallbladder that diffusely involved the entire gallbladder mucosa. Additional resection was performed, and R0 surgery was achieved with no recurrence 1 year after surgery without adjuvant therapy.

## DECLARATIONS

### Funding

The authors declare that they received no funding support for this study.

### Authors’ contributions

YK and M Hashimoto wrote the manuscript.

YK, RN, MS, and M Hashimoto were involved in the clinical management of the patient.

YK, M Hashimoto, KN, YH, and MS revised the manuscript.

All authors read and approved the final manuscript.

### Availability of data and materials

Not applicable.

### Ethics approval and consent to participate

Not applicable.

### Consent for publication

Informed consent was obtained from the patient for the publication of this report.

### Competing interests

The authors declare that they have no competing interests.
